# The relationship between pro-inflammatory cytokines and pain, appetite and fatigue in patients with advanced cancer

**DOI:** 10.1371/journal.pone.0177620

**Published:** 2017-05-25

**Authors:** Ørnulf Paulsen, Barry Laird, Nina Aass, Tor Lea, Peter Fayers, Stein Kaasa, Pål Klepstad

**Affiliations:** 1 Department of Cancer Research and Molecular Medicine, European Palliative Care Research Centre, Norwegian University of Science and Technology (NTNU), Trondheim, Norway; 2 Palliative Care Unit, Telemark Hospital Trust, Skien, Norway; 3 Edinburgh Cancer Research Centre, University of Edinburgh, Edinburgh, United Kingdom; 4 Faculty of Medicine, University of Oslo, Oslo, Norway; 5 Department of Oncology, Oslo University Hospital, Oslo, Norway; 6 Department of Chemistry, Biotechnology and Food Science, Norwegian University of Life Sciences, Ås, Norway; 7 Department of Public Health, University of Aberdeen, Aberdeen, United Kingdom; 8 Department of Circulation and Medical Imaging, Norwegian University of Science and Technology (NTNU), Trondheim, Norway; 9 Department of Anesthesiology and Intensive Care Medicine, St Olavs Hospital, Trondheim University Hospital, Trondheim, Norway; McGill University, CANADA

## Abstract

**Background:**

Systemic inflammation is associated with reduced quality of life and increased symptoms in patients with advanced cancer. The aims of this study were to examine the relationships between inflammatory biomarkers and the Patient Reported Outcome Measures (PROMs) of pain, appetite and fatigue; and to explore whether levels of baseline biomarkers were associated with changes in these PROMs following treatment with corticosteroids.

**Material and methods:**

An exploratory analysis was done on a trial examining the analgesic properties of corticosteroids in patients with advanced cancer. Inclusion criteria were: >18 years, taking opioids for moderate or severe cancer pain; pain ≥4 (numerical rating scale 0–10). Serum was extracted and levels of inflammatory biomarkers were assessed. PROMs of pain, appetite and fatigue were assessed using the European Organisation for Research and Treatment of Cancer Quality of Life Questionnaire-C30 (EORTC QLQ-C30). The relationships between PROMs and inflammatory biomarkers were examined using Spearman Rho-Rank and multiple regression analysis.

**Results:**

Data were available on 49 patients. Levels of sTNF-r1, IL-6, IL-18, MIF, MCP-1, TGF-β1, IL-1ra, and C-reactive protein (CRP) and Erythrocyte sedimentation rate (ESR) were elevated; IL-1β, IL-2, IL-4, IL-8, IL-10, IL-12(p70), interferon-γ, MIP-1α, and TNF-α were below the level of detection. The following correlations were observed: appetite and IL-6 and CRP; fatigue and IL-1ra (r_s_: 0.38–0.41, *p*< .01). There was no association between pretreatment biomarkers and effect from corticosteroid treatment.

**Conclusion:**

In patients with advanced cancer and pain, some pro-inflammatory cytokines were related to appetite and fatigue. Inflammatory biomarkers were not associated with pain or with the efficacy of corticosteroid therapy. Further research examining the attenuation of the systemic inflammatory response and possible effects on symptoms would be of interest.

## Introduction

Systemic inflammation has been described as the seventh “hallmark of cancer”[1]; necessary for tumorigenesis and maintenance and progression of the cancer state. Symptoms such as pain, cachexia, fatigue, cognitive impairment, anxiety and depression are common in patients either in isolation or in combination, termed symptom clusters. The biological basis for these symptom clusters has been postulated as being driven by the tumour-host interaction via the systemic inflammatory response [2] akin to cytokine-induced sickness behavior (a set of physiological and behavioral responses observed in sick individuals after the administration of infectious or inflammatory agents or certain pro-inflammatory cytokines) [3].

Cytokines are key mediators and provide homeostasis and immune control as part of the innate immune system through an intricate interplay with mutually dependent positive and negative feedback mechanisms [4]. In healthy individuals, equilibrium exists between pro- and anti-inflammatory cytokines. In advanced cancer, however, complex tumour-host interactions disturb this equilibrium. Data from patients with advanced cancer show a cytokine pattern indicating a state of simultaneous immunostimulation and immunosuppression. Pro-inflammatory cytokines predominate, finally resulting in increased concentrations of Macrophage Migration Inhibitory Factor (MIF), Tumour Necrosis Factor α (TNF-α), interleukin (IL) -6, IL-8, IL-10, IL-18, and Transforming Growth Factor β (TGF-β) in patients with advanced cancer [4].

Furthermore, study data have shown associations between serum concentrations of inflammatory biomarkers and symptoms in cancer patients. To illustrate, elevated level of C-reactive protein (CRP) was associated with pain, anorexia, dyspnoea, and fatigue in patients with cancer [5, 6]. In another study, increased serum concentrations of soluble receptor 1 for tumor necrosis factor (sTNF-r1) and IL-6 were related to an increase in the mean score for all 15 recorded symptoms, and in the five most severe symptoms, respectively, in patients with lung cancer undergoing concurrent chemoradiotherapy [7]. Schubert and coworkers found cancer related fatigue to be associated with elevated levels of biomarkers IL-6, IL-1 receptor antagonist (IL-1ra) and neopterin [8]. Two other studies found that increased levels of IL-6 were associated with major depression in patients with lung cancer [9] and pancreatic cancer [10]; the latter study also found an association between the cytokines IL-1β, IL-4, and IL-12(p70) and pain intensity, and between TGF-β and fatigue. Similarly, trials have indicated associations between inflammatory gene variants and several symptoms. For instance, in a study of lung cancer patients, gene variants for IL-8 and IL-10 were associated with pain, depressed mood and fatigue [11].

So far, the associations between symptoms and specific biomarkers have not been consistent, which may in part be due to use of cross-sectional study designs, inconsistency in measurements [12] and heterogeneous cancer patient populations. Despite these inconsistencies, the relationship between symptoms and pro-inflammatory cytokines remains of interest particularly in the light of recent work demonstrating the clear relationship between Patient Reported Outcome Measures (PROMs) of quality of life and the systemic inflammatory response [13]. Moreover, the relationship between individual pro-inflammatory cytokines and PROMs of pain, appetite and fatigue is of interest, especially in the setting of anti-inflammatory treatment with corticosteroids, which may have implications for the management of these symptoms.

In clinical practice, anti-inflammatory drugs are often used for symptom control [14]. In particular corticosteroids have been shown to improve appetite and fatigue in patients with advanced cancer [15–17], and data from clinical trials support the use of corticosteroids for 1–2 weeks for these indications [15, 18]. With regard to the treatment of pain, a randomized controlled trial found no analgesic effect of corticosteroids in cancer patients taking opioids [17], whereas two studies have showed an analgesic effect [19], or temporarily increased pain control [16]. A meta-analysis in a recently published Cochrane review concluded that corticosteroids could provide a mean reduction of pain intensity of 0.84 on a numeric rating scale (NRS 0–10) (confidence interval, (CI) -1.38 to -0.3) after one week of treatment [20]. The mechanisms of action are not well defined, but are thought to be mediated by their anti-inflammatory effects.

The current study was a secondary exploratory analysis of a biobank established as part of a randomized, controlled trial assessing the analgesic effects of methylprednisolone 32 mg daily in patients with cancer pain on opioids [17].

Therefore, the primary aim of this study was to examine the relationship between inflammatory biomarkers (cytokines and markers of the inflammatory response) and PROMs of pain, appetite and fatigue in patients with advanced cancer receiving opioids. A secondary aim was to explore whether levels of baseline inflammatory biomarkers were associated with changes in pain, appetite and fatigue following treatment with corticosteroids.

## Materials and methods

### Overall design

An exploratory analysis was undertaken on data from a randomized, double-blind, placebo controlled trial examining the analgesic efficacy of corticosteroids in patients with advanced cancer taking opioids [17]. Forty-nine patients were randomized to methylprednisolone 16 mg twice daily or placebo; 25 were evaluated in the corticosteroid arm, 22 were evaluated in the placebo arm. Thirteen patients randomized to placebo received corticosteroids on an open basis after the intervention period. PROMs from these patients were included in the analyses at follow up after corticosteroid treatment. Ethical approval was given for the primary study by the Regional Committee for Medical Research Ethics Central Norway (4.2007.846) and the Norwegian Directorate of Health; Clinical trial information NCT00676936, EudraCT No 2007-005617-19. Procedures were conducted in accordance with the Declaration of Helsinki, as revised in 1983. All patients provided written informed consent to their data being analysed in line with the present study.

Eligible patients met the following criteria: >18 years of age, taking opioids for moderate or severe cancer pain; cancer diagnosis; pain ≥4 (on a 0–10 Numerical Rating Scale (NRS)) at inclusion; expected survival > 4 weeks. Exclusion criteria included diabetes mellitus, peptic ulcer disease, and concurrent use of non-steroidal anti-inflammatory drugs (NSAIDs) [17].

Inflammatory biomarkers were assessed at baseline, i.e. before corticosteroid treatment. PROMs, were assessed at baseline and at follow up after 7 days of corticosteroid treatment using the European Organisation for Research and Treatment of Cancer—Quality of Life Questionnaire—C30 (EORTC QLQ-C30) [21]. The EORTC QLQ-C30 scores were calculated according to the EORTC scoring manual [22], scores range from 0 to 100; a higher score corresponds to a better health-related quality of life in the function scales (“better”), whereas a higher score represents a higher levels of symptoms (“worse”) in the symptom scales. The EORTC QLQ-C30 has been extensively validated in the cancer patient population and was completed between 08:00 and 17:00 hours in all study patients.

The inflammatory markers and cytokines selected for the present study included high sensitivity CRP, erythrocyte sedimentation rate (ESR), IL-1β, IL-1ra, IL-2, IL-4, IL-6, IL-8, IL-10, IL-12(p70), IL-18, interferon-γ, TGF-β1, MIF, TNF-α, Macrophage Inflammatory Protein-1α (MIP-1α), Monocyte Chemoattractant Protein-1 (MCP-1) and soluble Tumor Necrosis Factor receptor-1 (sTNF-r1). sTNF-r1 was chosen because it reflects TNF-α-activity, since TNF-α is among the most unstable cytokines [8, 23]. The cytokines were chosen on the basis of previous research on cancer related inflammation and symptoms [7, 24, 25]. The sera underwent one freeze-thaw cycle.

High sensitivity CRP analysis was performed at Fürst laboratories, Oslo. The cytokine analyses were performed at the Norwegian University of Life Sciences, Ås, using multiplex technology (Multiplex System, Bio-Rad Laboratories Inc., Austin, Texas) in which serum cytokine concentrations are measured in high-sensitivity assays. All samples were assayed in duplicate and the assays performed according to manufacturer’s instructions by laboratory personnel blinded to the rest of the data. Bio Rad Human Inflammation panels 6 plex kit containing IL-8, IL-12(p70), IL-2, IL-10, interferon-γ, and sTNF-r1; Bio Rad human group 1 and 2 9 plex kit containing IL-1β, IL-1ra, IL-4, IL-6, MCP-1, MIP-1α, TNF-α, IL-18, and MIF; and Bio Rad singleplex kit TGF-β1 were used. In one patient one of the parallels in the 6 plex kit showed very high values as compared to the other parallel (coefficient of variation: %CV 137–140 between the duplicates) and the other biomarkers from the same patient. This parallel was therefore excluded from the analysis. Except from this, no significant variation was noted between duplicates for any sample. The intra-assay CV was <10%. Cytokine / chemokine concentrations were interpolated from an appropriate standard curve. If the biomarker concentration was below the lowest point on the standard curve, we used the lowest value.

### Statistical analyses

As this was an exploratory analysis no formal sample size calculation was performed. Where appropriate, all data are reported as means with 95% confidence intervals (CIs), ranges, medians with interquartile ranges (IQRs), or frequencies. As the cytokines were not normally distributed, Spearman Rho-Rank was applied for the correlation analyses. Based on previous research [26], sex, BMI, and age were explored as possible confounding factors in a multiple regression model. Sex or BMI were associated (*p*< .05) with PROMs of fatigue, appetite, and dyspnoea, and physical and role function scales, but did not change the results (data not shown). Associations between pre-treatment inflammatory biomarker levels and changes in pain, appetite, and fatigue following corticosteroid use were explored using multiple regression analyses. Sex and BMI were included as covariates. In order to give some protection for multiple testing, a significance level was set to *p* = .01. SPSS v21.0 (Chicago, IL) was used for all statistical analyses.

## Results

Patient demographics, pain characteristics and analgesic use are shown in Table 1 (n = 49). The mean age was 63.9 years (CI: 61.2–66.8), mean Karnofsky Performance Status score (KPS) was 66 (CI: 62–70), median survival was 86 days (IQR: 39–197), mean body mass index (BMI) was 23.0 (CI: 21.6–24.5), and mean opioid consumption 259 mg / day (oral morphine equivalents) (CI: 178–339). Six patients received chemotherapy (Table 1). Data were available on 49 patients at baseline and on 38 patients at follow up having received methylprednisolone (n = 34), dexamethasone (n = 2) or prednisolone (n = 2). Mean dexamethasone equivalent dose was 5.5 mg/day [27].

**Table 1:** Demographics and medical characteristics of the study participants.

		Number of patients (n = 49)	Mean	Median	CI
**Sex**	Female	24			
	Male	25			
**Age**	Years		63.9	64.8	61.2–66.8
**Weight**	kg		68.3	67.0	63.8–72.9
**BMI**^a^	kg/m^2^		23.0	22.3	21.6–24.5
**Ethnicity**	Caucasian	49			
**KPS**^b^			66	70	62–70
**Survival (days)**			185	86	39–197 (IQR)^c^
**Cancer diagnosis**	Gastrointestinal	14			
	Lung	11			
	Gynaecological	10			
	Prostate	6			
	Breast	2			
	Other	8			
**Metastases**	Liver	17			
	Bone	15			
	Lung	7			
	CNS	2			
	Other	33			
	No metastases	2			
**Oral opioid dose mg/24h**	mg (OME)^d^		230	135	165–296
**Baseline opioid**					
	Morphine	15	185	80	58–312
	Oxycodone	19	148	110	98–198
	Fentanyl	13	368	420	215–522
	Other	2	459	459	-4198–5115
**Corticosteroid medication**		n = 38			
	Methylprednisolone	34			
	Dexamethasone	2			
	Prednisolone	2			
**Dexamethasone equivalent**	mg		5.5		Range: 1.5–8
**Concomitant disease**		29			
	Cardiovascular	18			
	Lung	7			
	GI/Hepatic	5			
	Other	18			
**Other drugs**					
	Peptic ulcer / Ulcer prophylaxis	19			
	Sleep medication	15			
	Anticoagulants	14			
	Acetylsalicylic acid	8			
	Cardiovascular	6			
	Statins	4			
	Hormones	3			
	Antidepressants	8			
	Antibiotics	1			
	Antifungal agents	3			
**Ongoing cancer treatment**					
	Radiotherapy	0			
	Chemotherapy	7^e^			
	Hormonal therapy	6			
	None	36			

^a^BMI: Body mass index,

^b^KPS: Karnofsky Performance Status Score,

^c^ IQR: Interquartile range,

^d^OME: Oral Morphine Equivalents,

^e^Paclitaxel (n = 1), Gemcitabine (n = 1), Pemetrexed (n = 1), Fluorouracil (n = 1), Fluorouracil and Irinotecan (n = 1), Fluorouracil and Leucovorin (n = 1), Capecitabine and Temozolomide (n = 1).

Mean PROMs at baseline (EORTC QLQ-C30 0–100) are shown in Table 2. Mean EORTC QLQ-C30 scores were above 65 points for pain, appetite and fatigue indicating severe symptom intensity. Role, physical, and social function and global health were below 45 points, indicating impairment in these function domains and health-related quality of life.

**Table 2:** EORTC QLQ-C30 measurements at baseline.

	Mean	Median	CI
**Physical function**	39.3	40	33.8–44.8
**Role function**	24.8	16.7	18.4–31.2
**Emotional function**	73.9	75.0	67.0–80.8
**Cognitive function**	68.8	66.7	60.7–76.8
**Social function**	44.1	50.0	35.5–52.7
**General health**	40.5	41.7	34.8–46.1
**Fatigue**	72.7	77.8	66.1–79.2
**Appetite loss**	68.0	66.7	59.3–76.8
**Pain**	78.9	83.3	74.1–83.7
**Nausea and vomiting**	31.0	16.7	23.0–39.0
**Dyspnoea**	47.6	33.3	39.1–56.1
**Sleep**	27.8	33.3	18.6–37.0
**Constipation**	46.5	50.0	34.8–58.3
**Diarrhoea**	22.2	0,0	13.0–31.4

CI: 95% confidence interval

Table 3 shows the median serum concentration of inflammatory biomarkers (cytokines, CRP and ESR) at study baseline. IL-1β, IL-2, IL-4, IL-8, IL-10, IL-12(p70), TNF-α, interferon-γ, and MIP-1α values were below the lower limit of detection. Median CRP and ESR were 44 and 42, respectively, and cytokines IL-1ra, IL-6, IL-18, MCP-1, MIF, sTNF-r1 and TGF-β1 were increased as evidence of systemic inflammation.

**Table 3:** Biomarkers and observed serum concentrations.

I	Concentration (pg/mL) median	Interquartile range (IQR)	LLOQ^a^
**CRP**	44	19.8–122.5	
**ESR**	42	18–83.8	
**IL-1ra**	21.7	21.7–126.8	21.7
**IL-6**	2.33	2.33–26.0	2.33
**IL-18**	103.2	73.4–164.3	1.1
**MCP-1**	64.1	46.9–107.3	1.5
**MIF**	134.9	85.4–334.2	4.8
**sTNF-r1**	10917	7223–15257	27.1
**TGF-β1**	45145	36714–52636	1.2

^a^LLOQ: Lower limit of quantification.

Table 4 shows relationship between biomarkers and pain, appetite and fatigue at study baseline. Moderate correlations were demonstrated between appetite and CRP and IL-6; and fatigue and IL-1ra (*r*_s_ = .38 - .41, *p* < .01). Pain was not significantly correlated with any biomarkers. For the other EORTC PROMs, low correlations were observed.

**Table 4:** EORTC QLQ-C30 measurements at baseline and correlations with cytokine serum concentrations.

	Fatigue	Appetite	Pain	Physical function	Role function	Emotional function	Cognitive function	Social Function	Quality of life	Nausea Vomiting	Dyspnoea	Sleep	Constipation	Diarrhea
**CRP**	0.26	0.38*	0.16	-0.55**	-0.89**	0.15	-0.18	-0.31	-0.33	0.13	0.28	0.22	0.35	-0.17
**ESR**	0.08	0.27	0.30	-0.47*	-0.53**	0.25	-0.15	-0.13	-0.32	- 0.03	0.19	0.01	0.31	-0.29
**sTNF-r1**	0.17	0.22	0.20	-0.55**	-0.52**	0.30	-0.11	-0.12	-0.31	0.02	0.17	0.05	0.15	-0.12
**IL-1ra**	0.41*	0.34	0.16	-0.38	-0.35	0.11	0.03	-0.21	-0.26	-0.06	0.34	0.16	0.13	-0.06
**IL-6**	0.20	0.41*	0.20	-0.51**	-0.59**	0.16	-0.19	-0.22	-0.20	0.10	0.29	0.21	0.27	-0.28
**MCP-1**	0.18	0.32	0.09	-0.20	-0.23	0.23	-0.14	-0.34	0.01	-0.14	0.06	0.07	-0.02	0.23
**IL-18**	0.13	0.28	0.15	-0.45*	-0.43*	0.28	0.10	-0.32	-0.12	-0.03	0.18	-0.05	-0.08	0.06
**MIF**	0.15	0.04	0.20	-0.33	-0.41*	0.24	-0.11	-0.06	-0.07	-0.17	0.22	0.03	-0.01	-0.15
**TGF-β1**	0.02	0.02	0.20	-0.19	-0.24	-0.25	-0.43*	-0.22	-0.29	-0.20	-0.16	0.39	0.17	0.14

* = *p*< .01,

** = *p*< .001 Blood samples for ESR (n = 1), CRP (n = 3) and for cytokines (n = 6) were missing.

For the EORTC function domains, strong correlations were found between physical function and CRP, IL-6 and sTNF-r1; and role function and CRP, IL-6, ESR, sTNF-r1 (*r*_s_> .50, *p* < .001). Moderate correlations were found between physical function and ESR, and IL-18; between role function and IL-18 and MIF; and between cognitive function and TGF-β1 (*r*_s_ = .40 - .50, *p* < .01).

Table 5 shows the relationship between serum concentrations of biomarkers at baseline and improvement in PROMs following treatment with corticosteroids. Serum-concentration of MCP-1 was correlated with pain intensity (β = -.38) (explained variability R^2^ = 0.13, *p* = .016), and sTNF-r1 was correlated with appetite (β = —.43) (explained variability R^2^ = 0.16, *p* = .012) after corticosteroid treatment, not significant when allowing for multiple comparisons.

**Table 5:** Fatigue, appetite and pain intensity and response to corticosteroid therapy.

	Fatigue			Appetite			Pain intensity		
	β	Sig	R^2^	β	Sig	R^2^	β	Sig	R^2^
**CRP**	0.07		.00	0.16		.02	-0.07		.00
**ESR**	-0.06		.00	-0.04		.00	-0.17		.03
**sTNF-r1**	-0.18		.03	-0.43	*p* = .012	.16	-0.34	*p* = .033	.10
**IL-6**	0.07		.00	-0.10		.01	-0.21		.04
**MCP-1**	-0.10		.01	-0.20		.03	-0.38	*p* = .016	.13
**IL-18**	-0.15		.02	-0.04		.00	-0.29		.08
**MIF**	-0.08		.01	-0.17		.03	-0.33	*p* = .034	.10
**TGF-β1**	-0.10		.01	-0.16		.02	0.02		.00

β = standardized beta. Multiple regression analysis: fatigue day 7 dependent; fatigue day 0, sex, and BMI as covariates. Biomarkers CRP, IL-6, IL-18, MCP-1, MIF, and TGF-β1 were log-transformed. Blood samples were missing for CRP (n = 3) and for cytokines (n = 6).

The relationships between individual inflammatory markers are shown in Table 6. Strong correlations were found between CRP and ESR, and IL-6; sTNF-r1 and IL-18, and MIF; IL-6 and IL-1ra, and MIF; and MCP-1 and IL-18, all correlations *r*_s_ > .50 (*p* < .001). A number of moderate correlations were observed *(r*_s_ = .39 - .50, *p* < .01).

**Table 6:** Correlations between the analysed cytokines.

	**CRP**	**ESR**	**sTNF-r1**	**IL-1ra**	**IL-6**	**MCP-1**	**IL-18**	**MIF**
**CRP**	1							
**ESR**	0.70**	1						
**sTNF-r1**	0.38	0.45*	1					
**IL-1ra**	0.32	0.28	0.21	1				
**IL-6**	0.69**	0.49*	0.37	0.55**	1			
**MCP-1**	-0.00	0.00	0.38	0.20	0.28	1		
**IL-18**	0.39*	0.41*	0.59**	0.49*	0.45*	0.52**	1	
**MIF**	0.31	0.40*	0.63**	0.32	0.54**	0.50*	0.31	1
**TGF-β1**	0.25	0.09	0.12	-0.12	0.16	0.15	-0.19	0.25

* = *p*< .01,

** = *p*< .001

## Discussion

The present study demonstrates that biomarkers of the systemic inflammatory response are related to appetite and fatigue in patients with advanced cancer with pain who are taking opioids. More specifically, decreased appetite was correlated with increased levels of IL-6 and CRP, and increased fatigue was correlated with increased IL-1ra. In contrast, pain was not correlated with any of the investigated biomarkers. No significant predictor for effect of corticosteroid treatment was identified.

The inflammatory biomarker panel included in the present study showed increased serum concentrations of IL-6, IL-8, MIF, sTNF-r1, and TGF-β1 in patients with advanced disease. Interestingly, this pattern corresponds to the cytokine pattern described in patients with advanced cancer by Lippitz [4]. It is also consistent with previous reports showing that systemic inflammation is related to multiple quality of life and symptom variables [6, 13]. In this study, IL-6 and CRP were related to deteriorating appetite, and animal studies have also proposed a link between loss of appetite and systemic and regional expression of the pro-inflammatory cytokines IL-1, TNF-α and IL-6 [28]. In other studies in patients with cancer, associations have been shown between appetite loss and IL-1β, IL-6 and IL-8 [29] and with gene polymorphisms coding for TNF-α [30], IL-1β [31], and IL-10 [32]. In our data, increasing IL-6 was the most prominent biomarker for appetite loss with explained variability R^2^ = 0.16.

The present study also observed a moderate correlation (*r*_*s*_ = .41) between fatigue and the anti-inflammatory cytokine IL-1ra. IL-1ra is a physiological inhibitor of IL-1β, its production is stimulated by IL-1β and IL-6 [33]. IL-1ra is expressed in higher concentrations in serum as compared to IL-1β, which has a short half-life and is degraded during storage [34]. Thus, IL-1ra serves as a marker for IL-1 activity [26, 35].

Work to date has suggested that cancer-related fatigue is linked to inflammatory, metabolic, neuroendocrine, and genetic biomarkers [12]. However, results for individual biomarkers are inconsistent which may be due in part to methodological issues [12]. In patients with advanced disease, several studies have shown an association between fatigue and CRP [6, 13, 36, 37]. However, in another trial this association did not persist after correction for covariates [38]. In one of the cited studies, IL-1ra and IL-6 were associated with fatigue in patients with advanced cancer [36]; although this was not found for IL-6, IL-1β, IL-8 or TNF-α in another study [29].

In patients with advanced cancer, intensity of fatigue has been associated with other symptoms, in particular pain, dyspnoea, anorexia, psychological distress, and insomnia [39]. Fatigue is commonly described in symptom clusters with pain [40, 41], and indeed the primary trial was a pain intervention trial. Our results showed that pain intensity was associated with fatigue, *r*_s_ = .38 (*p*< .01) (results not tabulated). In a regression model, pain and IL-1ra were both independently associated with fatigue, with an explained variability of R^2^ = 0.12 and R^2^ = 0.13, respectively. In three small clinical trials, IL-6 blockers [42, 43], and IL-1ra-treatment [44], were effective in decreasing disease activity and in alleviating fatigue when used for treating IL-6-mediated Castleman’s disease. Data also indicate that treatment with recombinant IL-1ra may alleviate fatigue in patients with rheumatoid arthritis and Sjögren’s syndrome [45, 46].

In the case of pain, the positive associations between pain and CRP [5, 6, 13] reported previously were not observed in the present trial. Moreover, intervention trials assessing effects of corticosteroids on cancer pain have also shown conflicting results. The primary trial found no evidence of an analgesic effect of methylprednisolone 32 mg daily for cancer pain [17]. Another trial found only a temporary effect of systemic corticosteroids on pain intensity [16, 47]. This is in contrast to a previous cross-over trial [19], in which 28 patients with predominantly bone localized pain (n = 16), visceral (n = 7) or nerve compression pain (n = 5) and low level of opioids showed response in pain intensity and analgesic consumption to methylprednisolone 32 mg daily. These observations suggest that cancer pain might be less associated with systemic inflammation than appetite and fatigue. However, the conflicting results may also suggest that there are subgroups of cancer pain that may respond better to corticosteroid treatment. Cancer induced bone pain might be such a subgroup. In this respect it is worth mentioning that patients with elevated pre-treatment serum-concentrations of MCP-1 were more likely to have an improvement in pain following treatment with corticosteroids (Table 4) (explained variability R^2^ = 0.13, *p* = .016, not significant when allowing for multiple comparisons). Correcting for the presence of cancer induced bone pain did not influence this result.

This observation corresponds to previous work that MCP-1 plays a role in chronic pain facilitation via its receptor, C-C chemokine receptor type 2 (CCR2) [48, 49]. Animal data show that MCP-1 expression in spinal neurons also is increased in animals with cancer induced bone pain. Moreover, MCP-1 induced hyperalgesia and anti-MCP-1 or CCR2 agonist attenuated hyperalgesia in animals with bone cancer when applied intrathecally [50, 51]. Furthermore, corticosteroids are shown to decrease MCP-1 [52]. Additionally, experimental animal studies suggest that locally applied sustained release methylprednisolone improved hyperalgesia in rats with compression radicular pain. This pain improvement was associated with a decreased number of infiltrating macrophages at the sciatic nerve, and reduced MCP-1 expression in the nerve [53]. In patients with cancer pain, MCP-1 was one of five cytokines that was significantly correlated with pain relief in a study on acute changes in cytokine serum concentrations during three hours of opioid treatment for pain [54]. Based on this basic science work, the observation that MCP-1 might be a biomarker for pain response from corticosteroids is interesting and should be tested in future studies.

A number of correlations were observed between biomarkers and EORTC function domains, in particular for deteriorating physical and role function which were associated with increasing CRP, IL-6, sTNF-r1, ESR, IL-18, and MIF. Multiple regression analyses showed that CRP was the most strongly correlated biomarker for role function, and IL-6 for physical function, with explained variability R^2^_adjusted_ = 0.34 and 0.28, respectively. Role function comprises two items, i.e. ability to perform work or to pursue hobby activity, while physical function items focus on physical capability and strength. Role and physical function items are closely related [55] and do probably express the same construct. Moreover, poor role function may also be related to high intensity of cancer-related fatigue in this cohort. We have identified two studies that report a multidimensional assessment of fatigue in patients with advanced cancer. These two trials observed associations with cytokines IL-1ra or IL-6, respectively, of the physical fatigue subscale only and not of the mental dimensions of fatigue [56, 57]. The EORTC fatigue item has been shown to correlate more strongly with the physical than the mental fatigue subscale of the Fatigue Questionnaire in palliative care patients [58]. Fatigue, but not pain intensity, was significantly associated with role function in our data, *r*_s_ = .54 (*p*< .001) (results not tabulated).

In our study, a correlation was also demonstrated between cognitive function, i.e. difficulty in concentrating and remembering things, and increasing serum concentrations of the anti-inflammatory cytokine TGF-β1. Data from patients with breast cancer suggest that IL-1β, IL-6, IL-8 and TNF-α contribute to chemotherapy-associated cognitive impairment [59]. Cognitive symptoms are frequent in patients receiving cytokine-based immunotherapies such as interferon-α and IL-2 [60]. However, an association between TGF-β1 and cognitive function is not previously described in results from clinical trials to the best of our knowledge.

In the multiple regression analyses, appetite was independently associated with IL-6 and CRP; and fatigue independently associated with IL-1ra. As for the EORTC function scales, only role function was independently associated with CRP and IL-6. These results support the clinical observations seen in the primary trial in which appetite and fatigue were statistically and clinically significantly improved after anti-inflammatory treatment with methylprednisolone [17]. Moreover, these observations also correspond to findings from another trial showing that dexamethasone improved fatigue and physical well-being [16]. Similarly, appetite, fatigue, and role function were the only EORTC QLQ-C30 items independently associated with systemic inflammation in patients with advanced cancer in a large, recent study [13]. Taken together, these data representing both cross-sectional data and intervention trials support systemic inflammation as a plausible causal factor in fatigue, reduced appetite, and impaired role functioning.

Persuasive arguments now support a move towards assessing the clinical usefulness of specific inhibitors of inflammation in treating or preventing symptoms caused by innate immune reactions in cancer. This type of studies will also provide further information regarding the possible role of cytokines in the pathophysiology of these symptoms. As for today, recombinant IL-1ra (anakinra) is one example of a viable therapeutic option, and intervention trials on IL-1ra administration for chronic fatigue syndrome are underway [61].

We recognize that the present study has some limitations. We included a limited number of patients, making the analyses susceptible to imprecise estimates and type II errors. In addition, we did not obtain blood samples after the intervention period and therefore we cannot compare PROMs with changes in cytokine concentrations after corticosteroid treatment. Another limitation is that this was a small sample with marked heterogeneity of the population studied, factors which may have influenced the findings. Also, the time of sampling was not strictly standardized. The diurnal variation of certain cytokines could influence the results as for instance IL-1, IL-6, TNF-α, and interferon-γ are linked to melatonin and have their peak early in the morning [8, 34].

It is also worth mentioning that as all patients in the study were taking opioids. The role of opioids in immune modulation is well documented [62, 63]. The immune modulatory effects may differ between opioids [63, 64]; and how they may affect the different cytokines is not well defined. Serum concentrations of cytokines were not associated with oral morphine equivalents in our study, and no differences were observed between morphine, oxycodone or fentanyl. However, sample size did not allow subgroup analyses. Additionally, as opioids may attenuate pain, and adverse effects may result in fatigue, all these factors may have influenced the findings.

However, to our knowledge, this is the first study assessing associations between inflammatory biomarkers and PROMs in the setting of an intervention trial with corticosteroids.

## Conclusion

Symptoms in patients with advanced cancer have been regarded as related to the underlying tumor bulk and its physiological consequences. However, the tumour-host interaction is likely to play an important role in symptom development and certain symptoms may be related to individual cytokines implicated in the pro-inflammatory response [65].

We report an association between inflammatory markers IL-6 and CRP and appetite, and IL-1ra and fatigue in cancer patients with advanced disease. Additionally, independent associations between role function and CRP and IL-6 were prominent. Whether or not these cytokines are responsible, in isolation or in conjunction with others, for the development or the progression of symptoms, remains unclear and is beyond the scope of the present study. However, the demonstration of the importance of systemic inflammation in the likelihood of responding to anti-cancer therapy [66], may be a paradigm that can be applied to symptoms. Our findings provide further weight to the argument that the systemic inflammatory response influences symptoms, specifically anorexia and fatigue, in cancer patients. Studies testing this hypothesis are needed and may have the potential to improve symptom control in patients with advanced cancer.
